# Modular megaprostheses yield high any-type failure rate but acceptable infection control rate in patients with chronic hip and knee periprosthetic joint infection and severe bone loss: a single-center experience

**DOI:** 10.5194/jbji-10-479-2025

**Published:** 2025-11-25

**Authors:** Vladislav Bartak, David Jahoda, Michal Benes, Tobias Judl, Matej Mazura, Petr Fulin

**Affiliations:** 1 1st Department of Orthopaedics, First Faculty of Medicine, Charles University and Motol University Hospital, Prague, Czech Republic; 2 Department of Anatomy, Second Faculty of Medicine, Charles University, Prague, Czech Republic

## Abstract

Chronic periprosthetic joint infections (PJIs) complicated by severe bone loss are challenging cases that require complex and specialized treatment approaches. Megaprosthetic replacement has gained in popularity in the setting of chronic hip and knee PJI; however, only a limited number of studies reporting on its utility are available. Thus, we aimed to review our cohort of patients with this specific condition who received modular megaprosthesis (MMP) as a limb salvage option in order to assess the failure rates, infection control, and implant longevity. We retrospectively reviewed electronic medical records of 61 patients who received MMPs for chronic hip and knee PJI between 2012 and 2024. The mean follow-up was 6.6±3.5 years. Failures were classified according to the Henderson classification. Kaplan–Meier survival curves were used to assess failure-free, infection-free, and overall implant survival. Cox regression analysis was performed to identify variables associated with MMP failure. Among the 61 patients, 37.7 % experienced any type of MMP failure, with infection recurrence being the most common reason for failure (60.9 %), followed by structural failure of the implant (17.4 %). At the 5-year follow-up, failure-free survival, infection-free survival, and revision-free survival were 65.8 %, 80.0 %, and 70.5 %, respectively. McPherson host grade C was significantly associated with implant failure (hazard ratio (HR) 3.1; 95 % confidence interval 1.4–7.6; P=0.024 ). Conclusively, MMPs represent a valuable treatment option for patients with chronic hip and knee PJI and large bone defects. While infection control is acceptable, the rates of any-type failure are high. These findings should be considered during preoperative patient counseling.

## 1 Introduction

Periprosthetic joint infections (PJIs) are dreaded complications following joint replacement surgery that are associated with increased patient morbidity and mortality (Ramos et al., 2025). Achieving infection control, particularly in the setting of chronic PJIs with multiple prior revisions, is often a complex and challenging task. Surgical treatment of such cases, which is regarded as the gold standard, is especially demanding due to a compromised soft-tissue envelope and, in particular, resultant loss of bone stock (Corona et al., 2018). Many of these cases still face infection recurrence and culminate in subsequent amputation of the affected limb (Sukhonthamarn et al., 2021).

The invention of modular megaprostheses (MMPs) has introduced novel solutions for managing large bone defects (Crimi et al., 2023). The utilization of modular off-the-shelf components allows individualized tailoring of the implant without the need for prolonged fabrication times of custom-made prostheses. While MMPs are routinely employed in oncologic patients following tumor resection, their application in revision orthopedic surgeries is progressively increasing in popularity (Lozano-Calderon et al., 2018). Recently, MMPs have gained widespread recognition in the management of significant bone loss in aseptic revision procedures and in the treatment of periprosthetic or complex fractures. Similarly, MMPs have emerged as a promising limb salvage option for patients with chronic PJIs who present with severe bone loss. Despite the initial skepticism regarding their applications in the context of bone and joint infection surgery, contemporary data suggest acceptable infection control results, yet the risk of MMP failure remains inherently high (Alvand et al., 2018; Artiaco et al., 2013; Corona et al., 2018). Nevertheless, the number of studies specifically addressing the application of MMPs in PJI management remains notably limited, which hampers the ability to draw reliable conclusions regarding their efficacy and long-term success in this specific setting (Sambri et al., 2023).

Therefore, in this single-institution study, we aimed to review our cohort of patients who underwent MMP implantation for chronic hip and knee PJI in order to assess the failure-free survival, including the analysis of variables associated with MMP failure, and to determine the infection treatment success rate and longevity of these implants.

## 2 Methods

**Figure 1:**
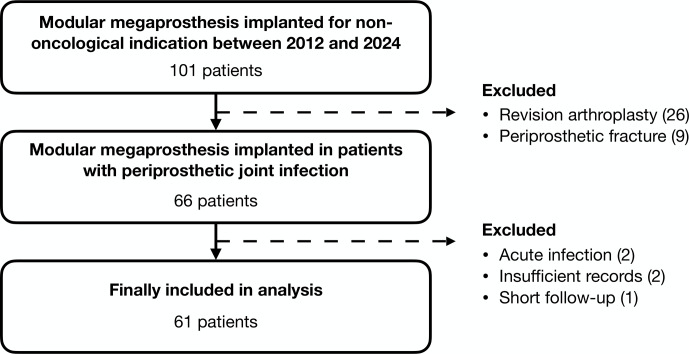
Flowchart outlining the inclusion of eligible patients.

Following institutional review board approval, we conducted a retrospective review of our prospectively maintained database of MMPs implanted for non-oncological indications to identify patients with chronic hip and knee PJIs where MMP was selected as a limb salvage option. All patients were treated at a single referral center with specialized musculoskeletal infection service between January 2012 and December 2024. Patients were included if they met the validated criteria outlined in the 2018 definition of periprosthetic hip and knee infection for the diagnosis of PJI (Parvizi et al., 2018), if PJI occurred more than 3 months since the last revision surgery (Parvizi et al., 2018), if they had a minimum follow-up period of 6 months, and if they possessed complete medical records. We identified 61 eligible patients meeting the inclusion criteria (Fig. 1).

### 2.1 Treatment protocol

All patients were treated in either two-stage or one-stage exchange regimes based on the individual situation and surgeon's preference. In the two-stage protocol, the original prosthesis was removed, followed by bone and soft-tissue debridement, irrigation (including pulse lavage), and the implantation of an articulating or static antibiotic-loaded spacer. At least five cultures, one sample for 16S PCR, and one or two histological samples were collected. The second stage consisting of MMP implantation was indicated once the patient was clinically found suitable and inflammatory markers improved. During the second stage, the spacer was removed and control cultures were collected, followed by additional debridement and implantation of the definitive MMP. The mean interval between stages was 4.0±2.6 months. None of the patients had positive cultures taken during the second stage.

**Figure 2:**
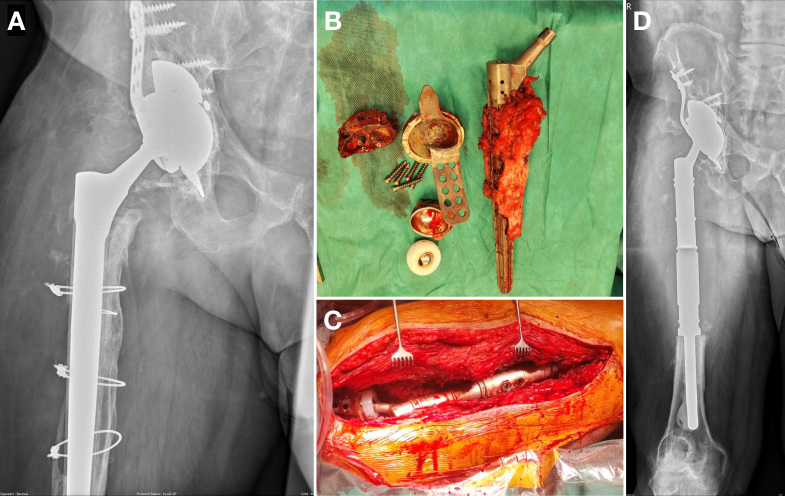
Illustrative case of one-stage exchange in a patient with 20 previous procedures. The preoperative x-ray shows bone destruction along the femoral stem **(a)**. Following implant removal **(b)** and thorough debridement, an MMP reconstructing two-thirds of the femur was implanted **(c, d)**.

One-stage exchange comprised original implant removal, thorough debridement, and irrigation with antiseptics and pulse lavage. Thereafter, wound-packing with antiseptic solution and temporary skin closure were performed, and the operating room was cleaned. Following instrument exchange, operative field preparation, and new sterile draping, the final MMP was implanted (Fig. 2). The same samples as in the two-stage exchange were collected during the initial phase and sent for testing.

The Modular Universal Tumor And Revision System (MUTARS^®^; Implantcast, Buxtehude, Germany) was used to reconstruct the femoral defects. At the acetabular side of the hip, either an ECOFIT^®^ 2M cup (Implantcast, Buxtehude, Germany) was used in cases of mild defects or a Trabecular Metal^®^ Acetabular Revision System (TMARS; Zimmer Biomet, Warsaw, USA) with an Avantage^®^ system (Zimmer Biomet, Warsaw, USA) was implanted in cases of larger defects. All patients received dual-mobility acetabular bearing. Cemented or cementless fixation was chosen upon the individual situation. Silver-coated MMPs were used according to the surgeon's preference. Some of the patients also received the local antibiotic-loaded carrier STIMULAN^®^ (Biocomposites Ltd, Keele, United Kingdom) at the time of MMP implantation, as it has been routinely used at our institution since 2020.

Antibiotic treatment was administered in cooperation with an infectious disease specialist, a microbiologist, and a clinical pharmacist. In general, following the first stage of the two-stage exchange, systemic antibiotics were maintained for a period of 4 to 6 weeks. Once the final MMP was implanted, systemic antibiotic treatment was resumed for another 2 to 6 weeks. In cases of microbiological findings of *Staphylococcus* species, rifampicin was added in combination. After one-stage exchange, patients with *Staphylococcus* species infections were treated with susceptible intravenous antibiotics combined with rifampicin for 2 to 6 weeks. After discharge, antibiotics were switched to peroral form for a total period of 3 months. In infections with other organisms, the total antibiotic treatment time was 4 to 6 weeks.

Patients were periodically followed up with clinical and radiographic examinations. Final follow-up data were derived from the last contact with our institution.

### 2.2 Data collection

A manual review of each patient's electronic medical record was performed to collect demographic data, physical status based on the American Society of Anesthesiologists (ASA) score and McPherson host grade (McPherson et al., 2002), microbiological information, number of previous surgeries, exchange regime, use of local antibiotic-loaded carrier and silver-coated prosthesis, length of modular components, and associated complications. Failures of MMPs were further classified according to Henderson et al. (2014), excluding Type 5 (tumor progression) and 6 (pediatric failure), which are not relevant for the purpose of our study. Infection recurrence was categorized either as relapse if the same microorganism was responsible for PJI after MMP implantation or as reinfection if a new microorganism was identified in the postoperative period (Corona et al., 2018).

### 2.3 Study outcomes

The primary outcomes of this study were to evaluate the failure-free survival, the infection treatment success rate, and the longevity of the MMP. Failure of the MMP was defined according to the Henderson et al. (2014) classification for failure of limb salvage. Treatment success of PJI was determined based on the international consensus meeting Delphi criteria (Diaz-Ledezma et al., 2013). The endpoint for MMP survivorship was established as operative revision with hardware removal or exchange. The secondary outcome of this study was to explore risk factors that are associated with MMP failure.

### 2.4 Statistical analysis

Continuous variables are presented as means ± standard deviations, and categorical variables are reported as raw numbers with percentages. Continuous variables were compared using an unpaired t test and a Mann–Whitney U test for parametric and non-parametric data, respectively. A $\chi^{2}$ test was used to compare categorical variables. Kaplan–Meier survival curves with 95 % confidence intervals (95 % CI) were constructed with any type of failure, infection recurrence, and MMP revision as endpoints. A log-rank test was used to compare survivorship differences. Cox regression was used to compare time-to-event outcomes while adjusting for age, sex, anatomic site, ASA score, McPherson host grade, presence of a high-virulence organism, modular component length, and number of prior procedures. For anatomic site as a variable, total femur replacement was assigned either to the proximal or distal femur group based on the original joint involvement. *Staphylococcus aureus*, *Enterococcus* species, beta-hemolytic *Streptococci*, gram-negative rods, and fungi were considered high-virulence organisms for analysis (Tarabichi et al., 2025). The level of statistical significance was set at P<0.05 . All analyses were performed using Prism v. 10.4.0 (GraphPad Software, USA).

### 2.5 Cohort characteristics

From the total of 61 patients included in the final analysis, there were 36 women (59.0 %) and 25 men (41.0 %), with a mean age at the time of surgery of 68.7±9.1 (range 37–82) years. A total of 22 patients (36.1 %) underwent proximal femur reconstruction, 36 patients (59.0 %) had distal femur replacement, and the remaining 3 patients (4.9 %) underwent total femur replacement. The mean follow-up duration was 6.6±3.5 years. All patients had multiply revised arthroplasty prior to the MMP implantation (Table 1). The most frequently isolated microorganism was coagulase-negative *Staphylococcus* (20 cases; 32.8 %), followed by methicillin-sensitive *Staphylococcus aureus* (7 cases; 11.5 %). Cultures were negative in 18 patients (29.5 %), despite clear signs of infection fulfilling the diagnostic criteria. Complete microbiological results are attached in Table 2.

**Table 1:** Characteristics of the entire cohort and of the success and any-type failure groups.

	Total	Success	Failure	P
	( n=61 )	( n=38 )	( n=23 )	
Age (years)	68.7±9.1	68.2±10.3	69.5±6.6	0.592
Sex				
Female	36 (59.0 %)	21 (55.3 %)	15 (65.2 %)	0.443
Male	25 (41.0 %)	17 (44.7 %)	8 (34.8 %)	
ASA score	2.4±0.6	2.4±0.6	2.5±0.5	0.643
McPherson host grade				
A	24 (39.3 %)	18 (47.4 %)	6 (26.1 %)	0.144
B	25 (41.0 %)	15 (39.5 %)	10 (43.5 %)	
C	12 (19.7 %)	5 (13.2 %)	7 (30.4 %)	
Anatomic site				
Proximal femur	22 (36.1 %)	15 (39.5 %)	7 (30.4 %)	0.745
Distal femur	36 (59.0 %)	21 (55.3 %)	15 (65.2 %)	
Total femur	3 (4.9 %)	2 (5.3 %)	1 (4.3 %)	
Number of previous surgeries	3.8±2.6	3.9±3.0	3.5±1.3	0.558
Exchange regime				
One-stage	15 (24.6 %)	11 (28.9 %)	4 (17.4 %)	0.309
Two-stage	46 (75.4 %)	27 (71.1 %)	19 (82.6 %)	
Local antibiotic carrier				
Yes	15 (24.6 %)	10 (26.3 %)	5 (21.7 %)	0.687
No	46 (75.4 %)	28 (73.7 %)	18 (78.3 %)	
Silver-coated prosthesis				
Yes	5 (8.2 %)	3 (7.9 %)	2 (8.7 %)	0.912
No	56 (91.8 %)	35 (92.1 %)	21 (91.3 %)	
Length of modular components (cm)	10.6±7.3	10.8±7.3	10.1±7.5	0.710
Follow-up duration (years)	6.6±3.5	6.3±3.7	7.2±3.3	0.371

**Table 2:** Overview of isolated microorganisms.

Microorganism	Number of
	patients
Coagulase-negative *Staphylococci*	20 (32.8 %)
*Staphylococcus aureus*	
Methicillin-sensitive	6 (9.8 %)
Methicillin-resistant	1 (1.6 %)
*Cutibacterium acnes*	3 (4.9 %)
*Enterococcus faecalis*	3 (4.9 %)
*Escherichia coli*	2 (3.3 %)
*Klebsiella pneumoniae*	1 (1.6 %)
*Serratia marcescens*	1 (1.6 %)
*Streptococcus agalactiae*	1 (1.6)
Polymicrobial	
*Streptococcus* species + *Haemophilus parainfluenzae*	5 (8.2 %)
*Enterococcus faecalis* + *Pseudomonas aeruginosa*	
Coagulase-negative *Staphylococcus* + *Staphylococcus lugdunensis* + *Corynebacterium tuberculostearicum*	
Methicillin-sensitive *Staphylococcus aureus* + *Enterococcus faecalis*	
Methicillin-sensitive *Staphylococcus aureus* + *Candida albicans*	
Culture-negative	18 (29.5 %)

## 3 Results

### 3.1 Failure-free survival and risk factors

**Figure 3:**
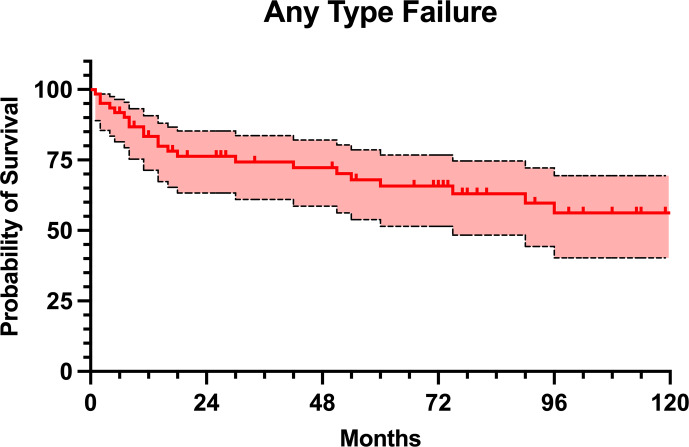
Kaplan–Meier survival curve with any type of failure as an endpoint.

A total of 23 (37.7 %) patients experienced any type of failure of the MMP, with a mean time to failure of 33.4±35.7 months (Table 3). The reasons for failure included 14 infections (Type 4; 60.9 %), four structural failures (Type 3; 17.4 %), three soft-tissue failures (Type 1; 13.0 %), and one aseptic loosening (Type 2; 4.3 %). Additionally, one patient developed an allergic reaction to nickel, requiring exchange for hypoallergenic implant (4.3 %). The failure-free survival rate was 83.4 % at 1 year, 76.3 % at 2 years, 65.8 % at 5 years, and 56.2 % at 10 years of follow-up (Fig. 3). Cox regression identified McPherson host grade > B as significant risk factor for failure (hazard ratio (HR) 3.1, 95 % CI 1.4–7.6; P=0.024 ), whereas age (HR 1.0, 95 % CI 0.9–1.0; P=0.326 ), sex (HR 1.2, 95 % CI 0.4–3.6; P=0.723 ), anatomic site (HR 1.0, 95 % CI 0.3–3.1; P=0.936 ), ASA score (HR 1.0, 95 % CI 0.4–2.8; P=0.944 ), presence of high-virulence organisms (HR 0.8, 95 % CI 0.2–2.4; P=0.635 ), length of modular component (HR 1.0, 95 % CI 0.9–1.1; P=0.620 ), and number of previous surgeries (HR 1.0, 95 % CI 0.7–1.2; P=0.804 ) were not significantly associated with failure.

**Table 3:** Descriptive data of patients with MMP failure.

Patient no.	Age	Sex	Anatomic site	No. of previous surgeries	Organism	Time to complication (months)	Specification	MMP retained	Final treatment
*Henderson type 1 – soft-tissue failure*									
1	59	F	Proximal femur	2	–	12	Dislocation	No	Cup exchange
2	71	M	Proximal femur	20	Polymicrobial	5	Dislocation	Yes	Closed reduction
3	61	M	Proximal femur	5	*Cutibacterium acnes*	1	Dislocation	No	Head exchange
*Henderson type 2 – aseptic loosening*									
4	76	M	Distal femur	4	–	16	Loosening	No	Exchange revision
*Henderson type 3 – structural failure*									
5	56	M	Distal femur	3	–	90	PE insert migration	No	Insert exchange
6	75	M	Distal femur	2	CNS	5	PE insert migration	No	Insert exchange
7	68	F	Distal femur	4	CNS	123	Periprosthetic fracture	Yes	ORIF
8	67	F	Distal femur	3	*Streptococcus agalactiae*	30	Periprosthetic fracture	Yes	ORIF
*Henderson type 4 – infection*									
9	64	M	Distal femur	5	CNS	14	Relapse	No	Knee arthrodesis
10	81	F	Distal femur	3	CNS	6	Quadriceps abscess	Yes	Drainage and lavage
11	73	F	Proximal femur	5	MSSA	8	Relapse	No	Girdlestone
12	66	F	Proximal femur	4	–	51	Relapse	No	Spacer
13	73	F	Distal femur	2	Polymicrobial	42	Relapse	No	Knee arthrodesis
14	80	F	Proximal femur	5	–	2	Relapse	No	Exchange revision
15	70	F	Distal femur	2	CNS	75	Reinfection	No	Exchange revision
16	74	M	Distal femur	4	MSSA	11	Reinfection	No	Knee arthrodesis
17	62	F	Distal femur	4	Polymicrobial	14	Reinfection	No	Knee arthrodesis
18	72	M	Proximal femur	2	*Cutibacterium acnes*	4	Relapse	No	Exchange revision
19	75	F	Distal femur	4	*Escherichia coli*	60	Relapse	No	DAIR
20	72	F	Proximal femur	4	MSSA	7	Relapse	No	DAIR
21	72	F	Distal femur	4	CNS	2	Relapse	No	Knee arthrodesis
22	70	F	Total femur	6	CNS	96	Relapse	Yes	Suppressive ATB therapy
*Other*									
23	65	F	Distal femur	3	CNS	93	Allergic reaction	No	Exchange revision

### 3.2 Infection-free survival

**Figure 4:**
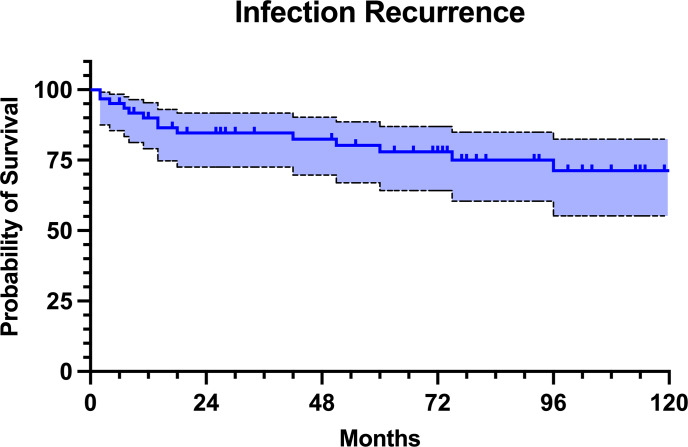
Kaplan–Meier survival curve with infection recurrence as an endpoint.

At the last follow-up, 47 patients (77.0 %) remained free of infection. Nevertheless, out of these patients, 9 (19.1 %) had revision surgery for aseptic reasons. The infectious complications were classified as relapse in 10 patients (71.4 %) and as reinfection in 4 patients (28.6 %). The infection-free survival was 90.0 % at 1 year, 84.6 % at 2 years, 80.0 % at 5 years, and 71.3 % at 10 years of follow-up (Fig. 4). Infection-free survival rates were similar in both proximal and distal femur replacement ( P=0.093 ). Additionally, there were no statistically significant differences in infection-free survival rates between one-stage and two-stage exchange regime ( P=0.711 ) or between patients who received a local antibiotic-loaded carrier compared to the ones who did not ( P=0.560 ).

### 3.3 Implant survivorship

**Figure 5:**
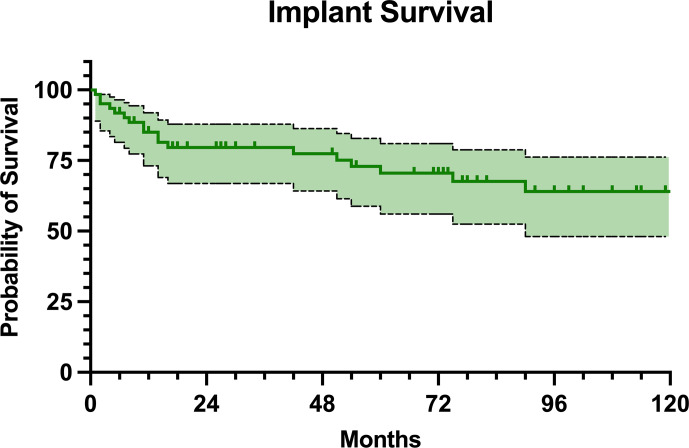
Kaplan–Meier survival curve with MMP removal as an endpoint.

Regarding the MMP survivorship, 43 (70.5 %) patients retained their implant at the final follow-up. The implant survival was estimated at 85.0 % at 1 year, 79.6 % at 2 years, 70.5 % at 5 years, and 64.0 % at 10 years of follow-up (Fig. 5). Only two of the patients with infection recurrence retained the implant (Table 3). No statistically significant differences in implant survival rates were found between proximal and distal femur replacement ( P=0.999 ).

## 4 Discussion

The management of chronic PJI remains one of the most formidable challenges in orthopedic surgery, especially when complicated by extensive bone loss and soft tissue damage. In view of the limited literature, our series contributes with insights into the role of MMPs as a salvage option in the context of complex and multiply revised infected hip and knee arthroplasties. This study demonstrated a relatively high rate of MMP failure at mid-term follow-up, with 37.7 % of patients experiencing any type of failure, corresponding to a 5-year failure-free survival rate of 65.8 %. However, only 14 of these patients developed infection recurrence, leading to a 5-year infection-free survival rate of 80.0 %. Given the results of our study, it is essential to recognize the high all-cause risk of failure of the MMPs when planning appropriate treatment strategy.

Besides the leading reason for MMP implantation in oncologic patients following tumor resection, non-oncologic indications include complex fracture, aseptic revision surgery, and PJI. Most of the existing studies combine the non-oncologic indications together, leading to only a few case series and retrospective reviews addressing solely the treatment outcomes in individuals with chronic PJIs (Alvand et al., 2018; Artiaco et al., 2013; Corona et al., 2018). In comparison with the existing literature utilizing MMP for management of patients with hip and knee PJI, the infection eradication rate of 77.0 % detected in our cohort is nearly similar to other studies. Alvand et al. (2018) reported an overall eradication rate of 72 % in 69 patients, and Corona et al. (2018) found an infection-free rate of 82.8 % in a series of 29 patients. Overall, slightly better numbers were reported for proximal femur replacement compared to distal femur replacement (Logoluso et al., 2022; Theil et al., 2022). However, our analyses showed no significant difference between the anatomic sites in terms of any type of failure, infection control rate, and MMP survivorship. These findings are in line with studies directly comparing outcomes between hip and knee PJI (Alvand et al., 2018). It must be mentioned that the surgical strategies and treatment protocols vary across the literature, along with the follow-up times which were shorter than in our study.

Studies comparing different indications for MMP implantation unanimously showed worse outcomes in patients with PJI (Grammatopoulos et al., 2016; Strony et al., 2022; Zajonz et al., 2016). Since the use of MMPs in the management of PJIs is associated with an increased risk of infection compared to native joints, there is a concern that larger implants may predispose to higher rates of infection recurrence (Theil et al., 2022). This is thought to be due to the increased surface area available for bacterial colonization and biofilm formation and to the complexity of eradication in these cases (Corona et al., 2018). Despite these concerns, the length of modular components was not found to be associated with MMP failure in our analysis.

While infection recurrence stands as the most common reason for failure, other complications have to be considered (Theil et al., 2023). In light of this fact, our study analyzed the risk factors for any type of failure. In our cohort, 37.7 % of patients experienced implant failure. Alvand et al. (2018) reported an even higher complication rate of 48 %. Across all studies, infection was responsible for approximately half of the failures. Generally, the most common aseptic complications are dislocations and periprosthetic fractures. These complications lead to the necessity of implant revision, which poses another treatment challenge because revision replacements were found to result in even higher failure rates compared to the first MMP implantation (Lozano-Calderon et al., 2018; Parikh et al., 2025). Generally, in our cohort, all patients who experienced any type of complication avoided amputation but were left with a varying degree of compromised limb due to additional revision, prosthetic extraction, or arthrodesis.

When looking at the variables in terms of different treatment modalities, there was no significant superiority. Silver-coated prostheses were believed to decrease the risk of infection recurrence (Fiore et al., 2023; Zajonz et al., 2017), but our findings supported by the recent literature do not suggest any significant impact in patients with PJI (Zajonz et al., 2016; Vicente et al., 2024). Nevertheless, a different scenario may be patients with oncologic disease in whom silver-coated prostheses appear to have better implant survival to infection (Pala et al., 2022). Similarly, there was no difference in infection-free survival between patients with and without local antibiotic-loaded carrier. Currently, there is no strong evidence suggesting the routine use of local antibiotics in the management of PJIs (Bourget-Murray et al., 2022).

Given the ongoing discussions regarding the optimal exchange regime (Corona et al., 2018), our sub-analysis comparing one-stage versus two-stage protocol showed no statistically significant difference in the infection recurrence-free survival. Two-stage exchange is especially demanding in cases with massive bone loss, since commercially produced spacers for such defects are not available worldwide. These circumstances require custom-made spacers, yet their fixation and stability are a major concern (Corona et al., 2018). Therefore, one-stage exchange appears to be suitable, alongside standard non-complicated septic revisions, in these demanding cases as well.

Our series includes a substantial percentage of culture-negative PJIs that were treated with MMP exchange. The high proportion of cases in which the causative pathogen was not identified may be attributable to several factors. The administration of empiric antibiotic therapy prior to the referral to our institution can suppress bacterial growth, thereby reducing the likelihood of pathogen identification in cultures. Moreover, the absence of routine implementation of next-generation sequencing (NGS) limits the ability to detect fastidious or non-culturable organisms that may be responsible for PJI. These factors have been demonstrated to significantly impair the sensitivity of conventional microbiological diagnostics, leading to a conclusion of culture-negative cases (Kalbian et al., 2020). Therefore, despite the standard use of PCR at our microbiology laboratories, implementation of other advanced molecular diagnostic techniques, such as the NGS, into the diagnostic workflow could potentially improve pathogen detection rates. All culture-negative cases in our series were treated as per protocol; thus the prophylactic antibiotics were maintained over the desired postoperative period. Nevertheless, although not tested, it seems that there is no significance in surgical decision-making and outcomes in these cases.

Limitations of our study include its retrospective nature, which is prone to potential selection bias. Furthermore, functional outcomes and patient-reported quality-of-life measures were not captured in our study. The procedures were performed by numerous orthopedic surgeons with varying experience but still specialized in musculoskeletal infections. It must be mentioned that this might have influenced the choice of exchange regime and the use of silver-coated prosthesis or antibiotic-loaded carrier, and the practice evolution over time likely influenced the outcomes. Despite these limitations, our study represents one of the largest series evaluating the utility of MMPs implanted in patients with chronic PJI, and it reports on the longest follow-up to date. Future prospective and multi-centric studies with larger cohorts are needed to validate our findings.

## 5 Conclusions

In conclusion, MMPs represent a viable limb salvage option for patients experiencing chronic PJI and severe bone loss, resulting from multiple failed revision surgeries. Based on our results, the implantation of MMPs in these challenging cases, combined with strict adherence to the principles of modern bone and joint infection surgery, may ensure acceptable infection control rates. Nevertheless, MMPs inherently carry a high risk of failure which must be cautiously considered in consultation with the patient. In particular, patients classified as McPherson host grade C, representing compromised hosts, were found to be at greater risk of experiencing implant failure, underscoring the importance of individualized risk assessment.
